# Diversity in EWAS: current state, challenges, and solutions

**DOI:** 10.1186/s13073-022-01065-3

**Published:** 2022-07-06

**Authors:** Charles E. Breeze, Jason Y. Y. Wong, Stephan Beck, Sonja I. Berndt, Nora Franceschini

**Affiliations:** 1 Division of Cancer Epidemiology and Genetics, National Cancer Institute, National Institutes of Health, Bethesda, MD USA; 2 UCL Cancer Institute, University College London, London, WC1E 6BT UK; 3 Department of Epidemiology, University of North Carolina, Chapel Hill, NC USA

## Abstract

Here, we report a lack of diversity in epigenome-wide association studies (EWAS) and DNA methylation (DNAm) data, discuss current challenges, and propose solutions for EWAS and DNAm research in diverse populations. The strategies we propose include fostering community involvement, new data generation, and cost-effective approaches such as locus-specific analysis and ancestry variable region analysis.

## Background

Epigenetics is a product of environmental and genetic effects and is influenced by multiple factors, including smoking, aging, and ancestry [1]. The most studied epigenetic modification is DNA methylation, which involves the addition of a methyl group to cytosines at cytosine-guanine dinucleotides (CpG sites) and is typically analyzed in large-scale epigenome-wide association studies (EWAS). CpG sites with differential methylation (also called differentially methylated positions or DMPs) in EWAS are the epigenetic equivalent of a single nucleotide polymorphism in genome-wide association studies (GWAS). Studies have shown low representation of diverse populations in GWAS, suggesting the need for more research across populations [2], but less attention has been paid to EWAS. Increasing diverse population representation in epigenomics is crucial for the interpretation of disease-associated loci, where epigenetic context may provide critical insight into underlying biological mechanisms [3]. 

## Population diversity in EWAS and DNAm research

Studies have reported notable population-specific DNAm differences [1, 4], showing that population diversity is critical to the interpretation of EWAS. However, the extent of population diversity in current DNAm data has not received sufficient attention. To highlight this issue, we summarize population diversity information from two large-scale publicly available databases for study-level EWAS metadata and individual-level DNAm data: EWAS Atlas and EWAS Data Hub, respectively [5, 6]. 

**Fig. 1:**
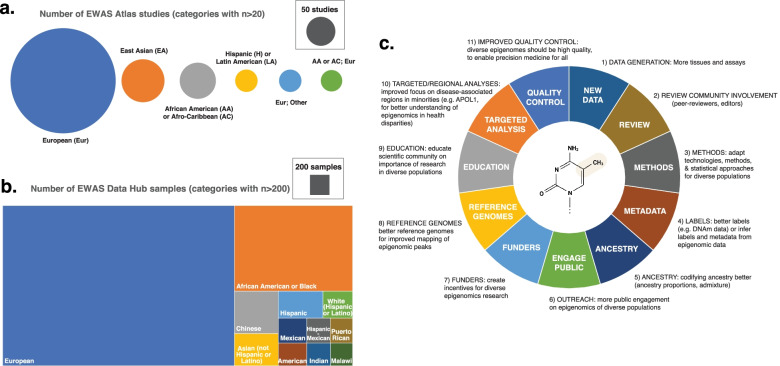
The state of diversity in EWAS data and future steps: Number of (**A**) studies and (**B**) individual-level samples across ethnicities. Study-level information was taken from EWAS Atlas (https://download.cncb.ac.cn/ewas/EWAS_Atlas_cohorts.tsv, accessed 4 Nov. 2021, taking ethnicity categories with over 20 samples). Individual-level sample data was taken from EWAS Data Hub (https://ngdc.cncb.ac.cn/ewas/datahub, accessed 4-Nov-21, taking ethnicity categories with over 200 samples. Ethnicities “White (not Hispanic or Latino),” “Caucasian,” “White,” “European American,” “Caucasian - European,” “Northern European,” “Utah, northern and western Europe,” and “European” were grouped into the European category. **C** List of future steps we propose to address current issues and ensure an appropriate roadmap for increasing value and diversity in EWAS and DNAm data. This list includes technical, scientific community and outreach-related steps. Only by including all players and aspects of scientific research will it be possible to fully realize the potential of epigenomic data in diverse populations

First, we analyzed the repository of study-level EWAS metadata (EWAS Atlas). Of the 1010 studies reporting race or ethnicity in EWAS Atlas (53.69% of total, accessed 4 Nov. 2021), most comprised individuals of European descent (Fig. 1A, *n* = 620 studies, 61.38%), followed by East Asians (*n* = 104 studies, 10.29%), and African Americans/Afro-Caribbeans (*n* = 74 studies, 7.32%). No other race or ethnicity comprised more than 5% of studies.

Regarding assays, in EWAS Atlas, the Illumina 450K DNAm array was the most frequently used assay (95.3% of studies with race/ethnicity information, *n* = 963) and hence had the highest degree of racial/ethnic diversity, with 9 ethnic groups included. The other assay, the more recently available Illumina 850K array, had less diversity with data for only 4 populations (European, East Asian, African American/Afro-Caribbean, and unspecified African), but much fewer studies reported using this technology (*n* = 19).

 Regarding biosamples, the range of different tissues/cell types was much greater for studies in Europeans, with 39 different tissues/cell types analyzed, including whole blood and different solid tissues, followed by East Asians (18 tissues/cell types) and African Americans/Afro-Caribbeans (11 tissues/cell types). All other races/ethnicities included in EWAS Atlas presented fewer than 10 cell types or tissues analyzed.

Second, we analyzed the database of individual-level DNAm data (EWAS Data Hub). Although, as previously noted, race and ethnicity information was only available for a subset of studies in EWAS Atlas (*n* = 1010, 53.69% of total), we noted similar racial/ethnic disparities in individual-level data reported in EWAS Data Hub (data reporting race or ethnicity at https://ngdc.cncb.ac.cn/ewas/datahub, accessed 4 Nov. 2021). Here, most individuals were in the European category (Fig. 1B, *n* = 14,630, 66.18%), followed by African American or Black (*n* = 3994, 18.06%), Chinese (*n* = 735, 3.32%), Asian (*n* = 560, 2.53%), and Hispanic (*n* = 472, 2.13%). Other major categories include Indian (*n* = 214, 0.96%) and Malawian (*n* = 200, 0.90%).

## Diversity and integrative analysis of EWAS

Disparities in chromatin mapping data generation between populations may generate biases impacting the functional interpretation of disease-associated loci. Chromatin mapping data can help to identify regulatory elements associated with EWAS loci in tissues and cell types relevant to disease etiology [7–9]. The extent to which current chromatin mapping resources, which are mostly European-centric, facilitate interpretation of EWAS loci in diverse populations is unknown.

Our prior work illustrated this issue in EWAS by applying an integrative epigenomic analysis method, eFORGE, on four DNAm meta-analyses of estimated glomerular filtration rate (eGFR): 1) European Americans (EA), 2) African Americans (AA), 3) Hispanic/Latinos (H/L), and 4) a combination of EA, AA, and H/L (transethnic analysis) [4]. Despite having a similar number of epigenome-wide associated loci when comparing EA and AA, enrichments in kidney regulatory elements—critical for a phenotype such as eGFR, where the kidney is a key player—were only detected for top EA CpGs, with much weaker results for other analyses [4, 10]. These findings may be the result of a lack of epigenetic data for non-EA populations, leading to a gap in the functional interpretation of study loci. Bridging this gap is especially critical given that conditions related to low eGFR disproportionately affect non-EA populations.

## Challenges and solutions regarding diversity in EWAS

More research is needed to explain observed epigenetic differences between populations. We have compiled a list of all the challenges regarding diversity in EWAS and propose some initial solutions to address them (Fig. 1C). One critical step is to generate more data in diverse populations to better understand health disparities, and make these data widely available. It is important to highlight the key role of the scientific community (peer reviewers and editors) to ensure that studies of diverse populations receive a fair review, given their contribution to novel insights in complex diseases, even though sample sizes may not always be as large as those from studies of European individuals. The strategy to implement this will likely involve collaborative efforts from multiple stakeholders including governmental organizations, academia, and industry. A consortium of multi-ethnic studies specifically focusing on increasing diversity in epigenomics (e.g., like the 1000 Genomes Project but for DNAm) would be of great benefit to the field. Frameworks like the International Human Epigenome Consortium (IHEC) or the Global Alliance for Genomics and Health (GA4GH) could help fund and structure these efforts. In any case, it will be important to ensure that appropriate local and international infrastructures are in place for storing, sharing, and perhaps even analyzing these diverse data. The focus should not only be to generate more blood-based analyses across ethnicities but also to generate in-depth analyses across other tissues in non-European populations, to better capture true epigenomic diversity. To increase awareness of this issue and provide appropriate incentives, grant review checklists should include a focus on data in diverse populations. Furthermore, a focus on DNA sequence-based ancestry metrics in addition to local race/ethnicity labels will improve comparability and robustness of analyses in diverse populations. The distinctions between race, ethnicity, and ancestry are complex, and educating the scientific community on these differences and the importance of diversity research is fundamental to future efforts. Involvement of the growing number of companies in the epigenomics and personalized medicine areas would be of great utility for diversity-increasing efforts. A version of the Personal Genome Project started by George Church that is specifically tailored towards epigenomics (e.g., Personal Epigenome Project) where users could donate personal DNAm data may provide an appropriate framework for individual contributions.

Importantly, new data must be generated in meaningful numbers to be able to obtain comparable sample sizes to European studies. Experimental design and quality control procedures must be implemented at a high and reproducible standard to truly make an impact given that technical factors and differences in DNAm data processing may obscure meaningful biological differences. In addition, certain future efforts may gain cost efficiency and power by focusing solely on ancestry-specific regions of DNAm variation, which can be profiled via targeted technologies (for example, via multiplexed targeted bisulfite sequencing approaches). For specific traits or diseases with a known genetic component, power may be gained by focusing on locus-specific analysis of regions surrounding population-specific genetic risk variants. For current databases, ancestry information can be dramatically expanded using recently developed tools to predict ancestry information from DNAm data [11].

In summary, there is a lack of diversity in currently available EWAS data, with most studies having been conducted in individuals of European descent. Increasing diversity in epigenomic data across populations has the potential to improve our understanding of disease risk, both for genomic loci with known population-specific genetic risk variants (e.g. APOL1) and for genome-wide interpretation of findings from EWAS. More efforts are needed to generate data in diverse populations using state-of-the-art approaches, in whole blood and in other primary tissues, to address important research questions regarding health conditions that disproportionally impact non-European populations.

## Data Availability

Study-level information was downloaded from EWAS Atlas (accessed 4 Nov. 2021, https://download.cncb.ac.cn/ewas/EWAS_Atlas_cohorts.tsv). Only unique studies were considered. Data used for the figure is publicly available at https://github.com/charlesbreeze/EWAS_Atlas/blob/main/EWAS_Atlas_cohorts.unique.csv.
